# Hypermixed Convolutional Neural Network for Retinal Vein Occlusion Classification

**DOI:** 10.1155/2022/1730501

**Published:** 2022-11-11

**Authors:** Guanghua Zhang, Bin Sun, Zhaoxia Zhang, Shiyu Wu, Guangping Zhuo, Huifang Rong, Yunfang Liu, Weihua Yang

**Affiliations:** ^1^Department of Intelligence and Automation, Taiyuan University, Taiyuan 030000, China; ^2^Graphics and Imaging Laboratory, University of Girona, Spain; ^3^Shanxi Eye Hospital, Taiyuan 030002, China; ^4^Department of Computer, Taiyuan Normal University, Jinzhong 030619, China; ^5^The First Affiliated Hospital of Huzhou University, Huzhou 313000, China; ^6^Shenzhen Eye Hospital, Jinan University, Shenzhen 518040, China

## Abstract

Retinal vein occlusion (RVO) is one of the most common retinal vascular diseases leading to vision loss if not diagnosed and treated in time. RVO can be classified into two types: CRVO (blockage of the main retinal veins) and BRVO (blockage of one of the smaller branch veins). Automated diagnosis of RVO can improve clinical workflow and optimize treatment strategies. However, to the best of our knowledge, there are few reported methods for automated identification of different RVO types. In this study, we propose a new hypermixed convolutional neural network (CNN) model, namely, the VGG-CAM network, that can classify the two types of RVOs based on retinal fundus images and detect lesion areas using an unsupervised learning method. The image data used in this study is collected and labeled by three senior ophthalmologists in Shanxi Eye Hospital, China. The proposed network is validated to accurately classify RVO diseases and detect lesions. It can potentially assist in further investigating the association between RVO and brain vascular diseases and evaluating the optimal treatments for RVO.

## 1. Introduction

Early changes in the retina are influenced by many factors, such as unfavorable environmental factors, including aging, a high-carbohydrate diet, and a sedentary lifestyle [1], and systemic diseases, including hyperglycemia [2], hyperlipidemia [3], and hypertension [4]. Retinal blood vessels are the only blood vessels available for noninvasive imaging in the human body. The pathological changes in retinal blood vessels occur much earlier than clinical symptomatic lesions. Therefore, retinal images have been widely used to detect early signs of systemic vascular diseases. In recent years, with an increase in the elderly population and the acceleration of the aging society in China, the fundus diseases of the elderly occur more frequently. Impaired vision can significantly impact an older person's quality of life and ability to live independently [5].

**Figure 1:**
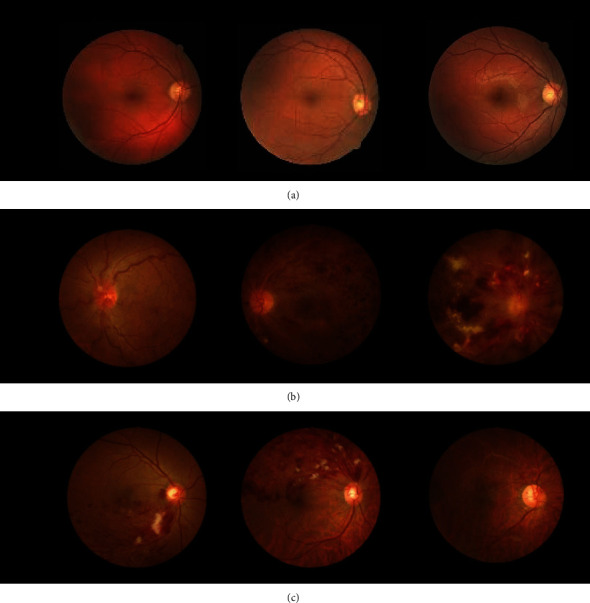
(a) Normal retina images, (b) CRVO retina images, and (c) BRVO retina images.

Retinal vessels are important structures of our eyes [6], and their detection and analysis are of great significance for the study of ocular diseases. Patients with retinal diseases may exhibit serious complications that cause severe visual impairment owing to a lack of awareness of retinal diseases and limited medical resources [7]. Retinal vein occlusion (RVO) [8] is one of the important eye diseases considered a risk factor for cardiovascular mortality and stroke in aging people [9]. Its typical symptoms include exudate [10], capillary nonperfusion [11], collateral formation [12], microaneurysm [13], sclerosed veins [14], and telangiectatic vessels [15]. Ischemic RVO is usually complicated by macular edema (ME) [16] and retinal and iris neovascularization [17], resulting in significant visual loss. RVO is classified into central and branch RVO (CRVO and BRVO). CRVO involves superficial or deep retinal hemorrhages (HEs) [18] that are scattered around the vein near the lamina cribrosa [19], and BRVO involves hemorrhages occurring within the occluded venule from the retinal sector to the blood supply sector, which is caused by arterial compression onto veins [20] (see Figure 1). RVO is the second most common retinal vascular eye disease after diabetic retinopathy (DR) [21]. If RVO is not treated in a timely manner, it can lead to serious complications that cause severe visual impairment [22, 23]. So far, the number of patients with RVO has increased [24], but our understanding of its pathogenesis, our ability to modify the final visual outcome, and the availability of treatments to effectively intervene in the progression of the disorder are all relatively limited [25].

Diagnosing ophthalmological diseases through deep learning models [26] has been used broadly in recent years [27]. A convolutional neural network (CNN) is one of the most famous deep learning architectures designed in 1989 [28]. Krizhevsky et al. [29] trained a large CNN architecture with eight layers and millions of parameters using a large ImageNet data set containing 1 million training images. In the field of ophthalmology, Litjens et al. [27] used a CNN to automatically segment macular edema based on OCT images. Google used a CNN network to automate the classification of diabetic retinopathy [30] and obtained the experimental results of 99% referral accuracy by training more than 100,000 data sets. The technology has been approved by the FDA as an official medical product. CNN is good at extracting feature information of different colors, spaces, and edges of images by using convolution modules of different scales and integrating all features into higher-order abstract features of images through continuous nonlinear transformation combinations. High-order abstract features and basic features are used together in the final learning process. CNN is almost a conventional method for medical image analysis, including color fundus images (CFIs) [27]. It is better in configuring spatial information by taking images as input. The achievements of CNN in autodiagnosis on different medical aspects can be found in [27], and it has been proved to surpass humans in some cases. So far, a hierarchical CNN architecture capable of distinguishing between normal CFIs and BRVO CFIs has been proposed by Zhang et al. [31] and developed by Zhao et al. [32]. Over the past several years, many modified and deeper CNN architectures have been proposed, which are not only used in the medical imaging domain but also widely applied in other domains [33].

Our study proposed an advanced model that can classify all normal, CRVO, and BRVO CFIs and detect the visible hemorrhage areas. It will help ophthalmologists realize computer-aided diagnosis in pathological analysis [34] to alleviate their pressure and discover and treat RVO as early as possible [35]. We will illustrate the methods employed in our model in the next chapter, followed by experimental results and final remarks in the third and fourth chapters, respectively.

## 2. Materials and Methods

**Figure 2:**
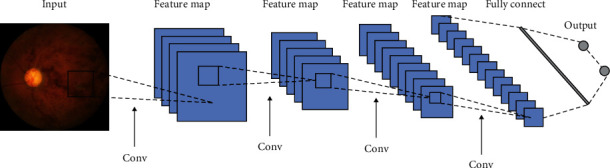
A simple CNN framework containing input, convolutional, pooling (subsampling), and fully connected layers (Heung-II, [36]).

When dealing with medical images, the structural and configuration information between adjacent pixels is a great source for analysis. A CNN that combines convolutional layers, pooling layers, and fully connected layers is more capable of extracting this type of information from 2D or 3D images. Convolutional layers apply a convolution operation by processing images only in receptive fields and adapting the weights gradually during the learning process. Pooling layers are usually followed by convolutional layers to reduce the dimensions of their output. Fully connected layers flatten data from previous layers to one dimension. A simple CNN framework is shown in Figure 2.

In this study, we also use the same data sets to conduct the control experiments on Resnet-34 [37], Inception-V3 [38], and MobileNet [39] models. Resnet [40] is the champion of the ImageNet large-scale visual recognition challenge (ILSVRC) in 2015. Resnet-34 [37] model is mainly composed of residual blocks, through which a deep network can be built and residual learning can be carried out in the feature extraction process. The Inception model is a deep CNN architecture proposed by Szegedy et al. [41] in ILSVRC 2014. The asymmetric multiconvolution kernel structure of the Inception-V3 [38] model performs the splitting operation on the larger convolution. Convolution kernels with different sizes are adopted so that receptive fields of different sizes can exist. The calculation efficiency of model parameters is improved, and the overfitting of the model is reduced. The MobileNet [39] model was proposed by a Google team [42] in 2017 and consists of a series of basic deep separable convolution (DSC) units. The model has a high precision and involves a small number of parameters and calculations.

### 2.1. Model Architecture

The study introduces a new CNN framework to classify RVO types and detect lesions. It is known as the VGG-CAM network, which utilizes a modified VGG19 network, general average pooling (GAP), class activation mapping (CAM), and CAM attention.

**Figure 3:**
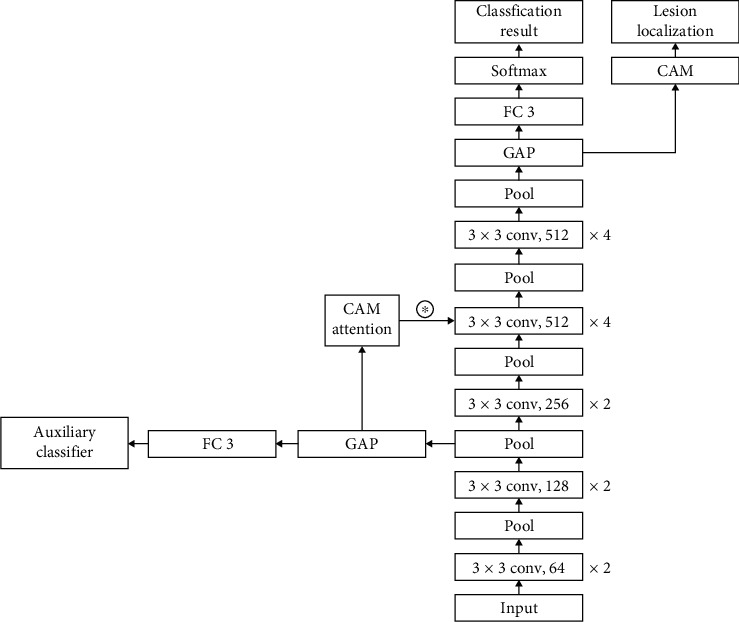
Framework of VGG-CAM model for RVO classification and lesion detection.

VGG19 [43] is a CNN architecture introduced by Simonyan and Zisserman. They used small receptive fields (3 × 3 matrix) to detect features from different positions of images and added the number of convolutional layers to increase the reception area for these receptive fields. Our VGG-CAM network reduces the number of fully connected layers in the original VGG19 networks from three to one and replaces them with a GAP layer. In the feature extraction stage, the auxiliary classifier and CAM attention layer are introduced to further enhance the model's activation weight for the lesion area. An additional CAM layer is connected with the GAP layer for lesion detection. SoftMax is applied as an activation function for the final fully connected layer, which predicts probabilities of different classes that the CFI can be labeled. The 24-layer framework of the VGG-CAM network is shown in Figure 3.

**Figure 4:**
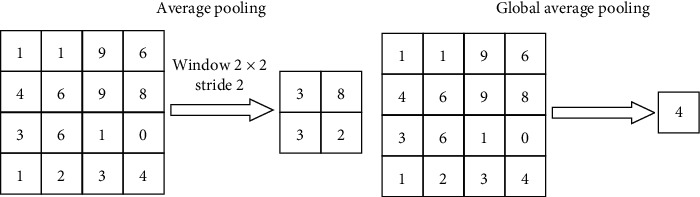
Examples of average pooling and GAP.

In the VGG-CAM network, the GAP layer preserves more information from input images [44], which helps detect lesion areas in the input image. Compared with average pooling, GAP only outputs one parameter from each receptive field (see Figure 4). Compared with the former, it uses less time to optimize the network. Lin et al. proved that the reduction of parameters in GAP has no effect on the accuracy of final networks [44].

**Figure 5:**
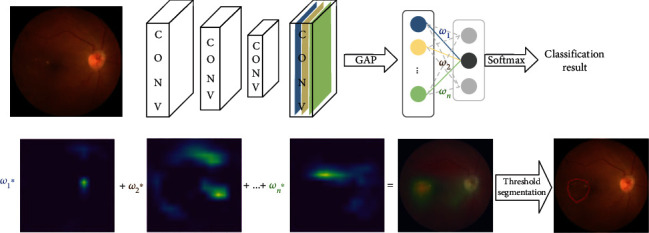
RVO lesion detection by CAM.

The CAM layer's computation is as follows:

(1) $$\begin{array}{c} \text{CAM}=\overset{C}{\underset{i=0}{\sum }}\omega_{i}*F_{i}, \end{array}$$

where *C* represents the number of channels in the feature map of the previous GAP layer. For each feature channel, the CAM layer augments products of weights *ω*_*i*_ from the fully connected layer and feature maps *F*_*i*_ before the GAP layer, as seen in Figure 5.

The CAM layer first uses Equation (1) to compute the class activation image of the original CFIs. It then applies bilinear interpolation to turn the class activation image into the size of the original image, followed by a threshold segmentation to detect the lesion location.

In the feature extraction stage, some useless information often affects the final classification accuracy; further, the extraction of key pathological features is the key to classification. The attention mechanism can guide the model to independently select the lesion area to be noticed. The feature weights are generated by introducing the auxiliary classifier and CAM attention layer after the sixth pooling layer. The feature weight is introduced into the feature map of the 10th layer to improve the model's attention and learning of the lesion area and further improve the final classification effect and the accuracy of lesion detection.

### 2.2. Image Preprocessing

**Figure 6:**
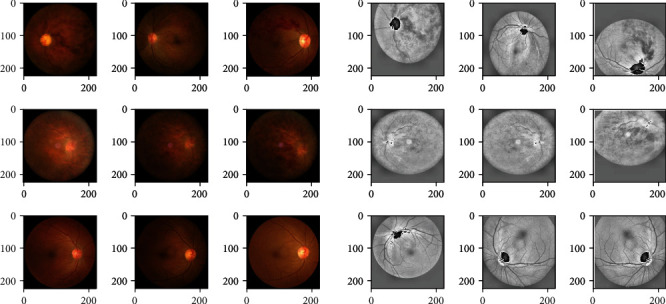
Input images before and after preprocessing.

Input images were mainly preprocessed by contrast limited adaptive histogram equalization (CLAHE) to increase contrast in original images [45]. Flipping, twisting, and zooming were used as well to increase the variety in our image database, which improves the model's ability to recognize various RVO images.  Figure 6 presents the differences between original and preprocessed input images.

### 2.3. Model Initialization

#### 2.3.1. Transfer Learning

Transfer learning [46] applies pretrained weights on a network from another problem as the initial weights for the same network in a different problem. In the problem of image processing, the shallow network of a neural network is mainly responsible for the feature extraction of shallow elements in an image, such as points, edges, and other such elements. Universal pretrained weights can reduce the network's learning time on a different problem [47]. The VGG-CAM model used pretrained weights from ImageNet as initial weights, which was trained by over a million images containing over 1,000 labeled images (https://www.image-net.org/).

#### 2.3.2. Stage-Wise Training

Stage-wise training [48] assigns priority to features of images. It separates the entire learning process into several sublearning processes, and the ability to extract different levels of image features is achieved through different learning processes. It allows information from the images to be processed gradually in the model [49]. In the earliest stage (the first eight layers in our model), the network accessed only a subset of the image, especially its coarse-scale features. Following stage II (8th to 13th layers) and stage III (13th to 18th layers), finer information was extracted from the image, and the feedback was used to evolve the previous stages for a better prediction. Stage III is the only prior for feature learning of the final stage (the fully connected layer).

#### 2.3.3. Environment

Operating system: Ubuntu 18.04 LTS; language: Python 3.6.8, Keras; GPU: GTX1080ti; CPU: Intel i7; Memory: Kingston DDR4 16 G.

## 3. Results and Discussion

### 3.1. Evaluation Metrics

In this experiment, the ability to identify unsupervised lesions was tested first, and then, the classification performance of the VGG-CAM model was tested in terms of sensitivity (Se), specificity (Sp), and Kappa. The calculation formulas of each index are shown below.

(2) $$\begin{array}{c} \text{Se}=\frac{\text{TP}}{\text{FN}+\text{TP}}, \end{array}$$

(3) $$\begin{array}{c} \text{Sp}=\frac{\text{TN}}{\text{FP}+\text{TN}}, \end{array}$$

(4) $$\begin{array}{c} \text{Kappa}=\frac{P_{0}-P_{e}}{1-P_{e}}. \end{array}$$

In Equations (2) and (3), TP indicates that the positive class is predicted as the positive class number, TN indicates that the negative class is predicted as the negative class number, FN indicates that the negative class is predicted as the positive class number, and FP indicates that the negative class is predicted as the positive class number.

In Equation (4), *P*_0_ represents the sum of the number of samples correctly classified for each class divided by the total number of samples, that is, the overall classification accuracy. Assume that the real number of samples of each class is *a*_1_, *a*_2_, ⋯, *a*_*n*_, respectively, the predicted number of samples of each class is *b*_1_, *b*_2_, ⋯, *b*_*n*_, respectively, and the total number of samples is *n*; then, *P*_*e*_ is expressed as

(5) $$\begin{array}{c} P_{e}=\frac{a_{1}\times b_{1}+a_{2}\times b_{2}+\cdots +a_{n}\times b_{n}}{n\times n}. \end{array}$$

### 3.2. Lesion Detection

**Figure 7:**
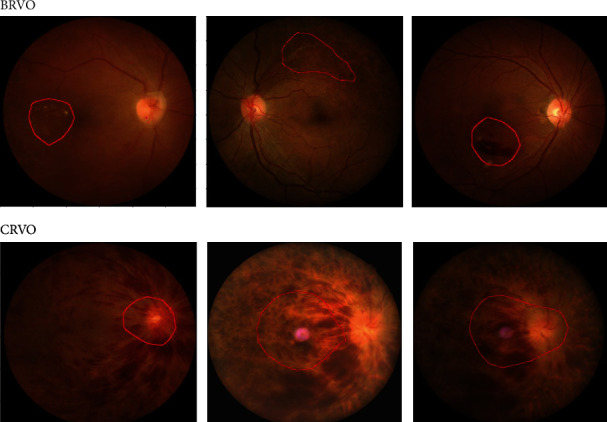
Lesion detection by VGG-CAM network on problematic areas of BRVO and CRVO.

Figure 7 presents examples of a detected lesion in BRVO and CRVO CFIs. For BRVO CFIs, the VGG-CAM network is sufficiently capable of identifying exudate, sclerosed veins, and hemorrhages. However, the network only highlights parts of a hemorrhage when the hemorrhage area is large. As for CRVO that has hemorrhage spreading all over the retina, the VGG-CAM network only indicates the central area of hemorrhage, namely, lamina cribrosa.

### 3.3. RVO Classification

**Figure 8:**
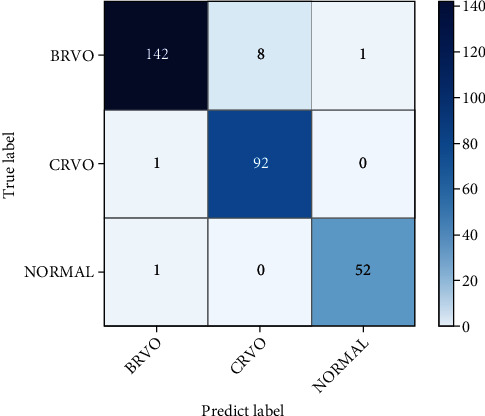
Confusion matrix of VGG-CAM network on validation set.

The performance of the VGG-CAM network on the validation set is shown in Figure 8 and Table 1. Model scores of the VGG-CAM network (see Table 1) show that the model has a high sensitivity and specificity in classifying BRVO, CRVO, and normal CFIs. When distinguishing between RVO and normal CFIs, the model has only one misclassified image. When distinguishing between BRVO and CRVO CFIs, it mislabeled eight CRVO images as BRVO. However, the sensitivity and specificity of classifying the three labels are above 94%, and the specificity is above 96%. The results of BRVO and normal CFIs are over 97% in the Kappa coefficient, but the results of CRVO CFIs are only 88%.

From the experimental results in Table 2, it can be seen that the sensitivity and specificity of the model after adding CAM and CAM attention layers are significantly improved when compared with the current classification models with better effects. From the results with CAM attention and without CAM attention, it can be seen that the CAM attention layer enables the model to more effectively extract lesion areas to enhance the final classification effect.

**Figure 9:**
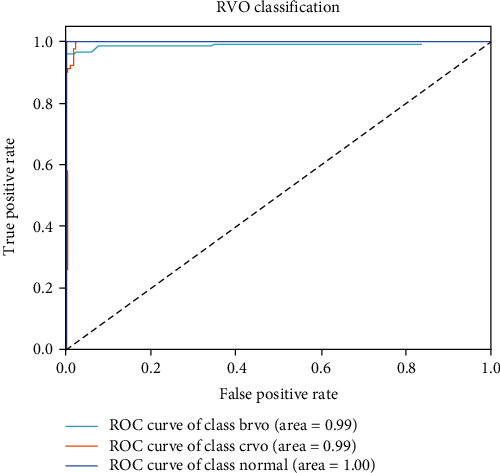
ROC curve of VGG-CAM network performance on RVO classification.

The following ROC curve (see Figure 9) plots the false positive rate (FPR) against the true position rate (TPR). The closer the curve is to (0,1), the more sensitive and accurate the model is. It shows that the area of all curves in the VGG-CAM model reaches 0.99, where the area under the curve of the normal label (1.00) indicates the model is capable of distinguishing between normal CFIs and RVO CFIs. The curves of the BRVO and CRVO labels have an area of 0.99, which indicates a slight probability of mislabeling between each other.

## 4. Conclusions

This study proposes a hybrid CNN, VGG-CAM, for RVO classification and lesion detection. The CAM attention layer was introduced to enhance the model's attention to the lesion area, and the network parameters learned from the ultralarge data set were used for the initialization of this network by migration learning. Stage training was used to reduce the training time of the model and improve the parameter optimization ability. Further, based on unsupervised learning method, the global average pooling and class activation methods were also used for lesion detection. The experimental results showed that the proposed model can accurately classify BRVO, CRVO, and normal fundus images, detect the lesion areas, and give the prediction results and clinical basis for the resulting judgment.

However, BRVO did not perform as well as CRVO and normal CFIs in sensitivity. For CRVO, the current lesion detection branches cannot achieve a high-precision prediction. This proposed model was only used for the preliminary study of fundus images in the field of view with a 55-degree lens. We concluded that the samples lacked diversity under the specific shooting field of the equipment. In future works, we will improve the model performance with image data from different medical devices and different fields of view and further improve the lesion detection accuracy of the model through a supervised learning method.

## Figures and Tables

**Table 1:** VGG-CAM network model scores on RVO classification.

Prediction	Sensitivity	Specificity	Kappa	Number of CFIs
BRVO	0.94	0.99	0.97	151
CRVO	0.99	0.96	0.88	93
Normal	0.98	0.99	0.98	53

**Table 2:** Comparison of the results of various methods on RVO classification.

Model	Sensitivity	Specificity
Resnet-34	0.92	0.92
Inception-V3	0.90	0.91
MobileNet	0.89	0.90
VGG-CAM without CAM attention	0.95	0.94
VGG-CAM attention	0.97	0.96

## Data Availability

The underlying data used to support the findings of this study are available from the corresponding author upon request.
